# DNA insecticides: Data on the trial in the field

**DOI:** 10.1016/j.dib.2018.11.016

**Published:** 2018-11-07

**Authors:** V.V. Oberemok, K.V. Laikova, N.V. Gal'chinsky, M.N. Shumskykh, A.I. Repetskaya, E.Yu. Bessalova, T.P. Makalish, Yu.I. Gninenko, S.A. Kharlov, R.I. Ivanova, A.I. Nikolaev

**Affiliations:** a Taurida Academy, V.I. Vernadsky Crimean Federal University, 295007, 4 Vernadsky Avenue, Simferopol, Crimea, Ukraine; b Medical Academy named after S.I. Georgievsky, V.I. Vernadsky Crimean Federal University, 295051, 5/7 Lenin Avenue, Simferopol, Crimea, Ukraine; c Botanical Garden named after N.V. Bagrov, V.I. Vernadsky Crimean Federal University, 4 Vernadsky Avenue, 29500 Simferopol, Crimea, Ukraine; d All-Russian Research Institute for Silviculture and Mechanization of Forestry, 15 Institutskaya Street, 141200 Pushkino, Russia; e Siberian Forest Research Station, 5a/2 Mechanization Street, 625017 Tyumen, Russia

## Abstract

This data article is related to the research articles entitled “The RING for gypsy moth control: topical application of fragment of its nuclear polyhedrosis virus anti-apoptosis gene as insecticide” (Oberemok et al., 2016), ”Molecular alliance of *Lymantria dispar* multiple nucleopolyhedrovirus and a short unmodified antisense oligonucleotide of its anti-apoptotic IAP-3 gene: a novel approach for gypsy moth control” (Oberemok et al., 2017), and “Topical treatment of LdMNPV-infected gypsy moth caterpillars with 18 nucleotides long antisense fragment from LdMNPV IAP-3 gene triggers higher levels of apoptosis in infected cells and mortality of the pest” (Oberemok et al., 2017). This data article reports on the significant decrease of survival of *L*. *dispar* larvae after contact application of 18 nucleotides long antisense oligoRING fragment in the field experiment and supports perspective of use of DNA insecticides in forests.

**Specifications table**

**Table:** 

Subject area	Biology
More specific subject area	Agriculture and forestry
Type of data	Graph
How data were acquired	*Lymantria dispar* larvae counting
Data format	Analyzed
Experimental factors	18 nucleotides long antisense oligoRING insecticide was applied topically
Experimental features	The experiment was conducted in the aspen forest on *Lymantria dispar* larvae in 2017
Data source location	Kulakovo, Tyumen Oblast, Russia
Data accessibility	All data are included in this document

**Value of the data**

• The data for the first time show that in the field experiment contact oligoRING insecticide is effective and decreases survival of *Lymantria dispar* larvae by 73.7% in 14 days in comparison with control group treated with water.
• The data on the absence of significant decrease of survival of *L*. *dispar* larvae in oligoHB group in comparison with water-treated control show that DNA insecticides are selective and effectiveness of their action depends on sequence of oligonucleotides in the fragment. Also, there was not found any visible abnormal tree growth and leaf color change in oligoHB and oligoRING-treated groups during the experiment and 1 year after treatment.
• The data show that applied concentration of DNA insecticides (1.2 micromoles per tree) is effective and can be affordable (synthesis of 1.2 micromoles of oligoRING insecticide costs around 0.2 US dollars).
• The data on the survival of *L*. *dispar* larvae after topical application of 18 nucleotides long antisense oligoRING fragment in the field experiment support the concept of contact DNA insecticides [1], [2], [3].

## 1 Data

**Fig. 1:**
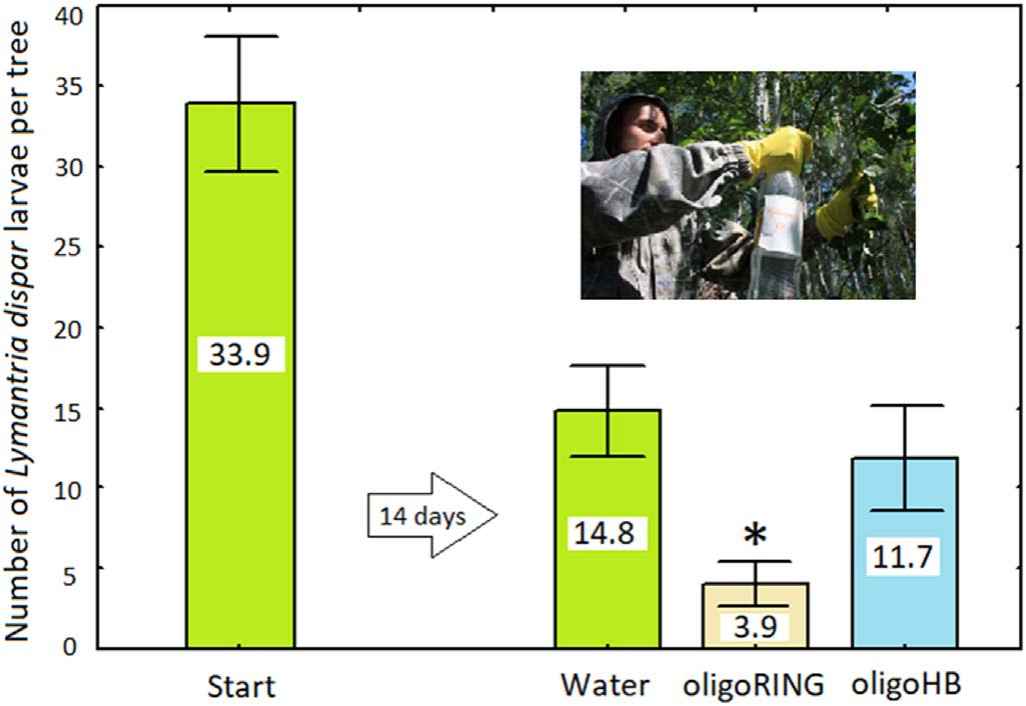
The survival of *Lymantria dispar* larvae after contact application of 18 nucleotides long antisense oligoRING fragment, control oligoHB fragment and water in the field experiment after 14 days; values represent means and standard errors; significance of difference versus water-treated control is indicated by * for *p* < 0.05.

Data for survival of *L. dispar* larvae after contact application of 18 nucleotides long antisense oligoRING fragment in the field experiment are represented in Fig. 1.

## 2 Experimental design, materials and methods

The experiment was conducted in the aspen forest near the village Kulakovo, Tyumen Oblast (Russia). 3 trees (2–3 m in height) with II-III instar *L*. *dispar* larvae were selected for each group of the experiment. 200 ml of water solution with a single-stranded oligoDNA fragment at a concentration of 5 pmol/µl (either oligoRING; 5′-CGA CGT GGT GGC ACG GCG-3′ or oligoHB sequence; 5′-GCT GCA CCA CCG TGC CGC-3′) was applied topically (1.2 micromoles of a DNA fragment per tree) using a hand-held sprayer at 23 °C. Control group was treated with water. *L*. *dispar* larvae counting was performed at the beginning (average number of larvae found for 9 trees) of the experiment and 14 days after the treatment. Statistical analysis was carried out in STATISTICA 7.0 using the Student׳s *t*-test.
